# Screening, diagnosing and treating deafness - the knowledge and conduct of doctors serving in neonatology and/or pediatrics in a tertiary teaching hospital

**DOI:** 10.1590/S1516-31802009000200002

**Published:** 2009-07-06

**Authors:** Patrícia Colozza, Adriana Ribeiro Tavares Anastasio

**Affiliations:** 1 Trainee in Speech Therapy at Hospital de Base, Faculdade de Medicina de São José do Rio Preto (Famerp), São José do Rio Preto, São Paulo, Brazil.; 2 MD, PhD. Audiologist and professor in the Department of Ophthalmology, Otorhinolaryngology and Head and Neck Surgery, Faculdade de Medicina de Ribeirão Preto (FMRP), Universidade de São Paulo (USP), Brazil.

**Keywords:** Questionnaires, Pediatrics, Hearing, Deafness, Early diagnosis., Questionários, Pediatria, Audição, Surdez, Diagnóstico precoce.

## Abstract

**CONTEXT AND OBJECTIVE:**

Infant hearing deficiency is a human disorder with devastating effects and serious implications for the development of speech and language. Early diagnosis of hearing loss should be the objective of a multidisciplinary team, and early-intervention programs should immediately follow this. The aim of this study was to investigate the knowledge and conduct of pediatricians and pediatric residents in a tertiary teaching hospital regarding deafness.

**DESIGN AND SETTING:**

Cross-sectional study in a tertiary hospital in the state of São Paulo, Brazil.

**METHODS:**

Eighty-eight questionnaires were randomly distributed to pediatricians and pediatric residents.

**RESULTS:**

Thirty-six questionnaires were analyzed. Most respondents (61.1%) were residents in pediatrics and/or neonatology. Eighty-three percent of them performed special procedures on babies presenting a high risk of deafness, and 55% reported that they had no knowledge of techniques for screening hearing. Most of them were unaware of the classifications of level and type of hearing loss. According to 47.2% of them, infants could begin to use a hearing aid at six months of age. Most of them reported that infants could undergo hearing rehabilitation during the first six months of life, and all respondents stated that being concerned about child communication is part of a doctor’s responsibilities.

**CONCLUSIONS:**

Even though most of the participants followed special procedures with babies presenting a high risk of deafness, they did not routinely investigate hearing. All respondents believed that it is a doctor’s responsibility to be concerned about child communication.

## RESUMO

**CONTEXTO E OBJETIVO:**

A deficiência auditiva na criança é uma das desordens humanas cujo impacto tem efeito devastador produzindo sérias conseqüências no desenvolvimento da fala e linguagem. O diagnóstico precoce da perda auditiva deve ser objetivo de uma equipe interdisciplinar e ser seguido imediatamente por programas de intervenção precoce. O objetivo foi investigar o conhecimento e condutas de pediatras e residentes em pediatria de um hospital-escola terciário em relação à surdez.

**TIPO DE ESTUDO E LOCAL:**

Estudo transversal, hospital terciário do Estado de São Paulo.

**MÉTODOS:**

Oitenta e oito questionários distribuídos para pediatras e residentes em pediatria.

**RESULTADOS:**

Analisados 36 questionários. A maioria dos entrevistados (61,1%) foi de residentes em pediatria e/ou neonatologia. Oitenta e três por cento realizavam alguma conduta especial com bebês de alto risco para surdez, e 55% referiram não ter conhecimento sobre técnicas de triagem auditiva. A maioria desconhecia as classificações de grau e tipo de perda auditiva. Para 47,2%, a criança poderia utilizar o aparelho auditivo a partir de seis meses. A maioria referiu que a criança poderia realizar reabilitação auditiva nos primeiros seis meses de vida e todos os entrevistados responderam que é função do médico ter preocupação com a comunicação da criança.

**CONCLUSÕES:**

Mesmo a maioria adotando condutas especiais com bebês de alto risco para surdez, não se investigava rotineiramente a audição. Todos os entrevistados julgaram ser função do médico se preocupar com a comunicação da criança.

## INTRODUCTION

Regardless of level, site or configuration, hearing deficiency in children is a silent, inconspicuous problem with serious consequences for the development of speech and language. It may lead to emotional, social and psychological problems, not only for the directly affected individuals but also for their relatives.^1^ For this reason, increasing concern about early identification of hearing abnormalities has led to the implementation of various neonatal hearing screening programs. The aim has been to test large numbers of babies by means of simple and rapid clinical procedures and, through this, to select and refer them for a more thorough evaluation when hearing abnormalities are suspected.^2^

The first years of children’s lives are a very important stage in their development. This is the phase during which behaviors and skills relating to social life develop, thereby providing indispensable elements for constructing self-perception and perception of the world.^3^

According to Resolution 01/99 of the Brazilian Committee on Hearing Loss in Childhood, the Universal Neonatal Hearing Screening (Triagem Auditiva Neonatal Universal, TANU) program aims to evaluate all neonates and is to be considered effective when at least 95% of all babies are evaluated. If it is impossible to implement TANU, the recommendation is to prioritize neonates that are at risk of deafness and gradually to extend the service to all newborns. In addition, among the diseases that can be screened at birth, hearing deficiency is a highly prevalent condition (phenylketonuria 1:10,000, hypothyroidism 2.5:10,000, sickle-cell anemia 2:10,000 and deafness 30:10,000).^4^ The incidence of significant bilateral hearing loss among healthy neonates is estimated as one to three per 1000 births and around 2% to 4% among infants from intensive care units.^4^ It has been estimated that 7% to 12% of all neonates have at least one risk factor for hearing deficiency.^5^

Over the last few years, early detection and treatment of hearing loss have gained great importance in pediatric practice,^6^ and pediatricians play a key role in the interdisciplinary teams that work to prevent hearing deficiency.^5^

Neonatologists, nurses and pediatricians are the first professionals that come into contact with neonates. Therefore, they are the ones who should determine whether or not neonates should be considered to be at high risk, by taking a brief history of the course of pregnancy and applying a questionnaire to the mothers.^7^

TANU is already mandatory according to municipal laws in several locations in Brazil^8^^,^^9^ and must be performed by hospitals, clinics and maternity hospitals.

Teaching hospitals are constantly engaged in the promotion of teaching, research, care and interdisciplinary practice. In order to rate interdisciplinarity among different healthcare fields, studies have been conducted in which questionnaires were applied to pediatricians and/or pediatric residents in order to determine their knowledge and conduct regarding questions involving hearing^5^^,^^10^^,^^11^^,^^12^ and speech therapy.^13^^,^^14^^,^^15^

## OBJECTIVE

The objective of this study was to investigate the concepts and conducts of pediatricians and pediatric residents in a tertiary teaching hospital regarding deafness.

## METHODS

The study was conducted at the Teaching Hospital of the Faculdade de Medicina de Ribeirão Preto (FMRP), Universidade de São Paulo (USP), Brazil.

Between April and July 2006, questionnaires were distributed to the team of male and female doctors in neonatology and/or pediatrics of the Pediatrics and Child Rearing Department of FMRP. This questionnaire, as proposed by Barros, Galindo and Jacob^11^ and adapted by the present authors, contained 11 multiple-choice questions and five yes/no items. Whenever the answer chosen was yes, respondents were required to describe their conduct. The questionnaires (Appendix 1) were distributed in the pediatric ward or at the pediatric outpatient clinic, and the professionals were instructed to return them within three days. Both the goals of the study and the procedures for filling out the questionnaire were explained at the time when it was distributed. The questionnaire consisted of written questions that were supposed to be answered individually, away from the researcher. In order to avoid disturbance to the clinical routine, participants were required to return the completed questionnaires to the office of the Pediatrics and Child Rearing Department of FMRP, from where the researcher would periodically collect them. Each question in the questionnaire was subjected to descriptive analysis, in percentage terms.

## RESULTS

Out of the 88 questionnaires distributed, 36 (40.9%) were completed and returned for analysis.

The respondents’ mean age was 34.4 years, and the mean time elapsed since their graduation was 9.9 years. Regarding their positions, 22 (61.1%) were residents, eight (22.2%) were professors, two were attending doctors, two (5.5%) were adjunct doctors, one (2.7%) was a trainee (2.7%) and one was a postgraduate student.

Thirty-one respondents (86.1%) reported that they worked in hospitals, one (2.7%) in a private practice, 19 (52.7%) in the public sector, and three (8.33%) elsewhere.

Most respondents (97.2%) reported that they had had training in hearing deficiency or deafness in undergraduate classes, two (9%) in specific courses and four (18%) in other settings such as congresses, postgraduate courses, self-study or personal experience.

The risk indicators for deafness, as reported by the respondents, are listed in Table 1. Thirty participants (83.3%) reported that they followed special procedures with high-risk babies. Twenty-seven of them (75%) said that they referred babies during the first six months of life, and one (2.7%) at the end of the first year. Two participants (5.5%) did not answer this question.

All participants agreed that it is possible to evaluate a baby’s hearing during the first six months of life; 19 of them (52.7%) were unaware of the possible techniques for evaluating infant hearing, and 20 respondents (55.5%) were unaware of hearing screening techniques.

When asked if they routinely investigated their patients’ hearing and at what age that was done, 19 participants (52.7%) answered “no”, and 17 (47.2%) answered “yes”. Sixteen of the latter group (44.4%) performed the investigation during the first six months of the baby’s life. The procedures reported by those who investigate their patients’ hearing include otoacoustic emission, use of the voice, finger snapping, physical examination, use of rattles, toys and hand clapping, asking the mother if the baby is frightened by noises, the brainstem auditory evoked response (BAER) test, a diapason (tuning fork) and a drum.

Thirty-one respondents (86.1%) reported that they did not know any classifications based on the level of hearing loss, and the others (13.9%) incorrectly rated hearing loss. Twenty-three participants (63.8%) did not know the different types of hearing loss, 11 (30.5%) reported that they knew them and two (5.5%) did not answer this question. Among those who reported that they knew the types of hearing loss, only one (2.7%) classified them correctly.

When asked about when they would refer children to a specialist for evaluation, 29 participants (80.5%) replied that they referred children when they were at high risk of hearing loss, 25 (69.4%) when they notice something unusual during their own evaluation, 20 (55.5%) when the mother made a complaint and four (11.1%) stated that they routinely referred children. Most respondents (97.2%) stated that they referred children for hearing assessment by a specialist during the first six months of life, and one (2.7%) stated that he would refer a child after reaching the age of three years.

When asked about the age at which a child could start using a hearing aid, 17 participants (47.2%) replied that it would be possible to do so after reaching six months of age, 13 (36.1%) that it would be possible at the end of the first year and four (11%) replied that that it would be possible at two years of age.

Twenty-four respondents (66.6%) said that a child could start hearing rehabilitation during the first six months of age, seven (19.4%) stated that it would be possible to start this at the end of the first year, two (5.5%) said that it would be possible during the second year of life, two (5.5%) stated that they did not know and one (2.7%) did not answer the question.

All respondents stated that doctors have a duty to be concerned about child communication.

**Table 1.** Indicators for risk of deafness considered by the respondents

Indicators for risk of deafness	% of respondents
Congenital infections	97.2
Bacterial meningitis/viral encephalitis	86.1
Ototoxic medication for more than five days	80.6
Craniofacial anomalies or congenital syndromes	77.7
Birth weight of less than 1500 g	77.7
Family history of childhood hearing deficiency	72.2
Neonatal suffering	61.1
Severe neonatal septicemia	55.6
Hyperbilirubinemia	47.2
Mechanical ventilation for more than 10 days	41.6

## DISCUSSION

Although the present study was conducted in a hospital devoted to teaching, research and care, less than half of the 88 questionnaires distributed (40.9%) were returned for analysis. Low adherence to this type of study has also been reported in a similar investigation.^5^ The most frequent explanation for failure to complete the questionnaire was lack of time, and some respondents stated that they were not interested in participating.

Most of the participants were medical residents working in hospitals. Thus, we believe that the mean age, time elapsed since graduation and the positions held by participants were strongly affected by this. In the literature that we consulted, the mean age and time elapsed since graduation,^10^^,^^11^ or the positions held by the respondents^5^ were not considered.

Thirty-five participants (97.2%) reported that they had had training in hearing or hearing deficiency during their medical courses. Some authors^5^^,^^10^ have pointed out that, in clinical pediatric practice, doctors’ knowledge about language development and hearing seemed to be limited, since most of the questions in their studies concerning these matters were not answered adequately. In the present study, most of the respondents mentioned different indicators of deafness risk (Table 1), which indicates knowledge of them in clinical pediatric practice. We emphasize here the importance of knowledge of the risk factors, since previous studies have shown associations of two or even three risk factors, thus implying significant hearing deficits.^2^^,^^14^^,^^16^^,^^17^ In addition, several diseases that can be detected in the nursery are responsible for childhood hearing deficiencies.^6^

Regarding babies at high risk of deafness, 75% of the respondents reported that they referred these babies for hearing evaluation during the first six months of life, although most of them were unaware of the hearing screening techniques. This result is a source of concern since, in many Brazilian municipalities, universal hearing screening is legally required^8^^,^^9^ and therefore should be known to most medical professionals, especially pediatricians.

In this regard, we consider that cooperation between pediatricians, audiologists and otorhinolaryngologists is essential for dissemination of hearing screening procedures, because pediatricians are the primary source of information and reference for parents regarding the development and health of their children. Awareness of the importance of these procedures for early diagnosis of deafness and information about the development of language and hearing are fundamental.

In contrast to other studies,^5^^,^^11^ our respondents claimed that it was possible to evaluate an infant’s hearing during the first six months of life. We consider this to be promising, since it demonstrates doctors’ concern regarding the consequences of deafness for child development, thus emphasizing the notion that early diagnosis allows for a more effective process of language acquisition and development.^18^ Prevention of deafness costs much less than its treatment. In addition, rehabilitation of these children to develop their hearing and integrate them into society is much more expensive and laborious for the healthcare sector than prophylaxis alone.^17^

Referral for hearing evaluation for infants at risk of deafness was the approach mentioned by 80.5% of the participants. However, even in the absence of a risk indicator, 64.4% of the participants who noticed something during medical evaluation referred the baby to a specialist. In 1994, the Joint Committee on Infant Hearing (JCIH)^19^ emphasized the need to identify all babies with hearing loss, and not only those with an indicator showing a risk of deafness. If this were not done, the use of such indicators to select babies for evaluation would mean that 50% of the infants with sensorineural hearing loss would not be identified. On this basis, universal neonatal hearing screening should be a priority.

Cury^20^ has drawn attention to the fact that pediatric textbooks contain little information regarding hearing deficiency and its possible causes. We noted that 97.2% of the respondents reported that they had had training in hearing and hearing deficiency at medical school. Most of the present respondents (86.1%) were unaware of the classifications of level and type of hearing loss. We believe that this knowledge would allow pediatricians to understand how the different types and levels of hearing loss interfere with the acquisition and development of speech and language and thus to identify different signs and symptoms that might occur among children.

In contrast to other studies,^5^^,^^10^^,^^11^ 47.2% were aware of the fact that it is possible to adapt to the use of a hearing aid starting at six months of life. We believe that our participants had better knowledge of this subject because the present study was conducted in a tertiary hospital that provides an accredited service of medium and high complexity hearing healthcare within the Brazilian national health system (*Sistema Único de Saúde*, SUS).

As noted in other studies as well, all of the respondents believed that doctors have a responsibility to be concerned about infant communication.^5^^,^^10^^,^^11^^,^^12^ Most of them stated that it is possible to perform hearing rehabilitation during the first six months of life. This emphasizes the credibility of rehabilitation for deaf children’s lives and increases rehabilitators’ responsibility within the field of hearing health.

At the time of distributing and collecting the questionnaires, the physicians expressed interest in having teaching and illustrative material produced on this topic. Recognition that certain limitations exist and an interest in bridging the many gaps makes it possible to exchange this important information among the various healthcare fields. We believe that acceptance of this could be the first step towards greater interaction between audiology and pediatrics, since our results demonstrated that the respondents had only partial knowledge of the evaluation, early diagnosis and treatment of deafness.

We argue that this situation can be changed if audiologists participate in the training of medical students and pediatric residents, by providing information regarding the development of hearing and the importance of early diagnosis aimed at clinical treatment.

Beyond being aware of the actions taken by pediatricians and neonatologists when faced with hearing deficiency in infancy, audiologists should strive towards wide dissemination of the importance of well-established cooperation among the professionals who deal with mother-child health, with emphasis on the importance of stronger participation in scientific events and scientific publications in related fields.

## CONCLUSIONS

Based on the analysis of the questionnaires, we conclude that:

1. Most of the participants identified one or more indicators for the risk of deafness but were unaware of screening techniques and hearing evaluations for children;
2. Most of the respondents followed special procedures for babies at high risk of deafness but did not routinely check the hearing of their patients and were unaware of the classification of hearing loss in terms of type and level;
3. All respondents believed that doctors have a responsibility to be concerned about child communication;
4. Most of the participants stated that it was possible to perform hearing rehabilitation for deaf infants during the first six months of life.
